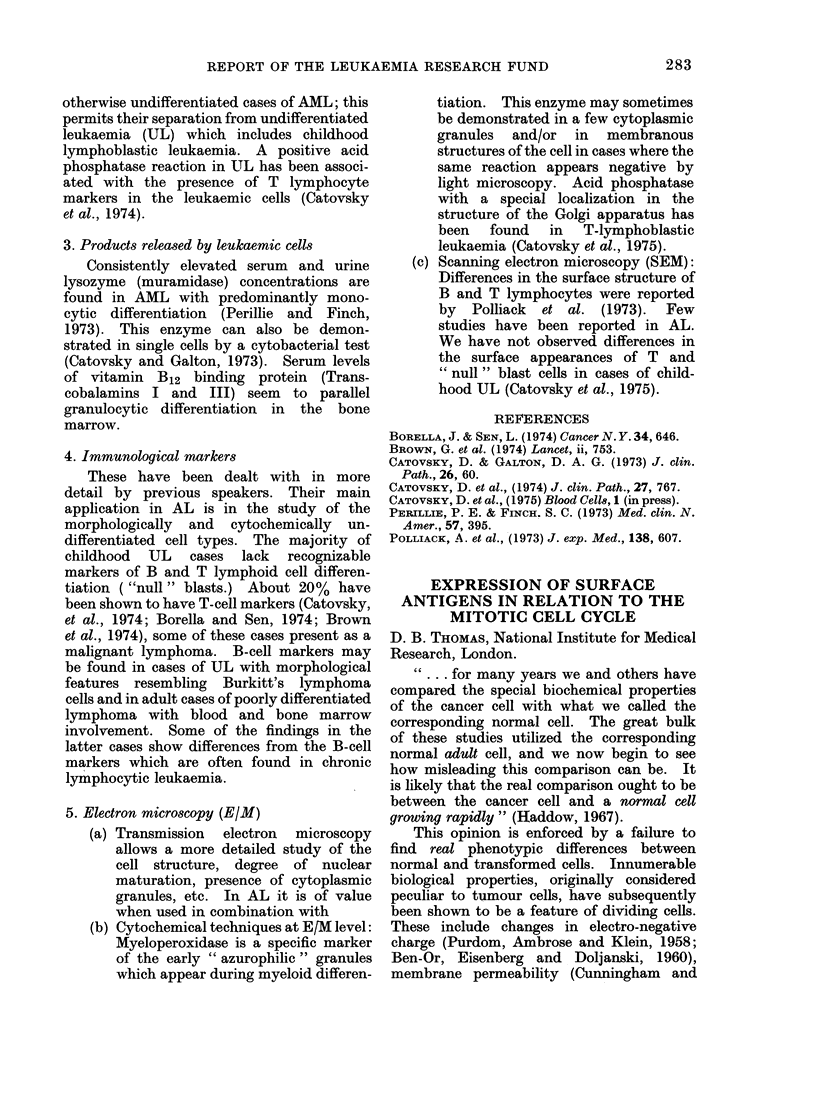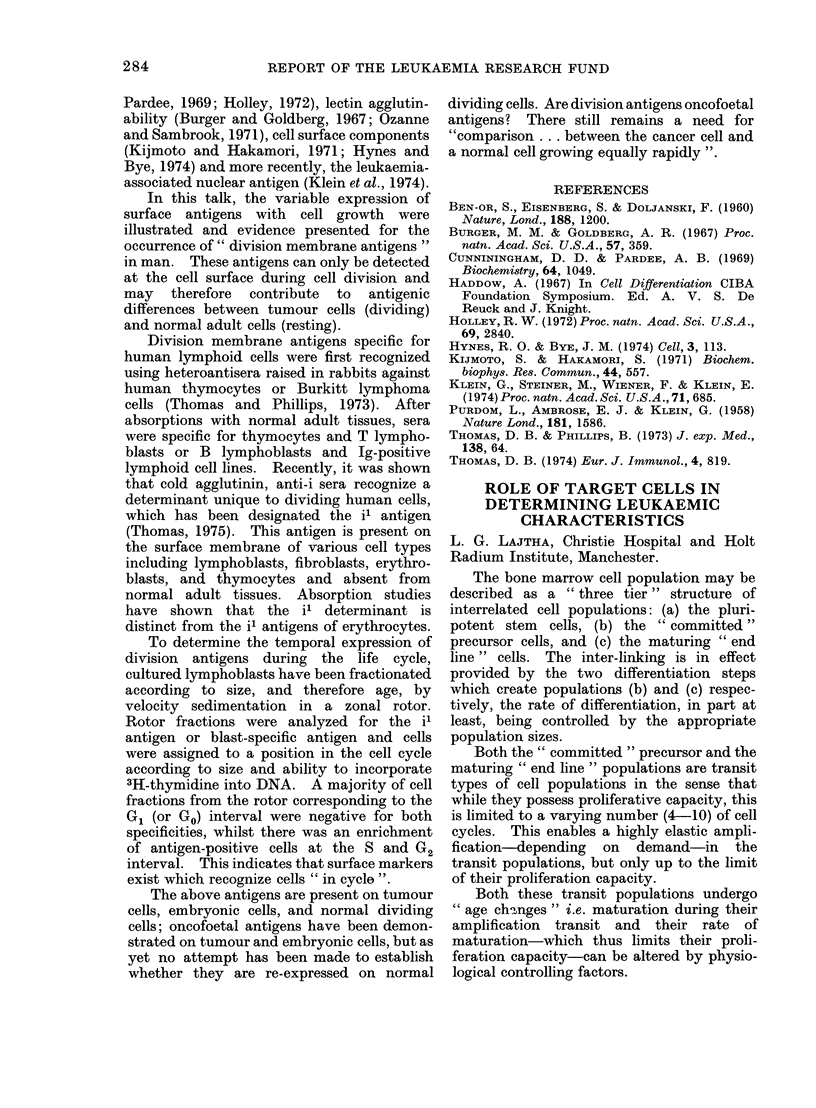# Proceedings: Expression of surface antigens in relation to the mitotic cell cycle.

**DOI:** 10.1038/bjc.1975.221

**Published:** 1975-08

**Authors:** D. B. Thomas


REFERENCES

BORELLA, J. & SEN, L. (1974) *Cancer N.Y.* **34**, 646.
BROWN, G. *et al.* (1974) *Lancet*, ii, 753.
CATOVSKY, D. & GALTON, D. A. G. (1973) *J. clin. Path.*, **26**, 60.
CATOVSKY, D. *et al.*, (1974) *J. clin. Path.*, **27**, 767.
CATOVSKY, D. *et al.*, (1975) *Blood Cells*, **1** (in press).
PERILLIE, P. E. & FINCH. S. C. (1973) *Med. clin. N. Amer.*, **57**, 395.
POLLIACK, A. *et al.*, (1973) *J. exp. Med.*, **138**, 607.

### EXPRESSION OF SURFACE ANTIGENS IN RELATION TO THE MITOTIC CELL CYCLE

D. B. THOMAS, National Institute for Medical Research, London.

"... for many years we and others have compared the special biochemical properties of the cancer cell with what we called the corresponding normal cell. The great bulk of these studies utilized the corresponding normal *adult* cell, and we now begin to see how misleading this comparison can be. It is likely that the real comparison ought to be between the cancer cell and a *normal cell growing rapidly*" (Haddow, 1967).

This opinion is enforced by a failure to find *real* phenotypic differences between normal and transformed cells. Innumerable biological properties, originally considered peculiar to tumour cells, have subsequently been shown to be a feature of dividing cells. These include changes in electro-negative charge (Purdom, Ambrose and Klein, 1958; Ben-Or, Eisenberg and Doljanski, 1960), membrane permeability (Cunningham and

Pardee, 1969; Holley, 1972), lectin agglutinability (Burger and Goldberg, 1967; Ozanne and Sambrook, 1971), cell surface components (Kijmoto and Hakamori, 1971; Hynes and Bye, 1974) and more recently, the leukaemia-associated nuclear antigen (Klein *et al.*, 1974).

In this talk, the variable expression of surface antigens with cell growth were illustrated and evidence presented for the occurrence of " division membrane antigens " in man. These antigens can only be detected at the cell surface during cell division and may therefore contribute to antigenic differences between tumour cells (dividing) and normal adult cells (resting).

Division membrane antigens specific for human lymphoid cells were first recognized using heteroantisera raised in rabbits against human thymocytes or Burkitt lymphoma cells (Thomas and Phillips, 1973). After absorptions with normal adult tissues, sera were specific for thymocytes and T lymphoblasts or B lymphoblasts and Ig-positive lymphoid cell lines. Recently, it was shown that cold agglutinin, anti-i sera recognize a determinant unique to dividing human cells, which has been designated the $i^1$ antigen (Thomas, 1975). This antigen is present on the surface membrane of various cell types including lymphoblasts, fibroblasts, erythroblasts, and thymocytes and absent from normal adult tissues. Absorption studies have shown that the $i^1$ determinant is distinct from the $i^1$ antigens of erythrocytes.

To determine the temporal expression of division antigens during the life cycle, cultured lymphoblasts have been fractionated according to size, and therefore age, by velocity sedimentation in a zonal rotor. Rotor fractions were analyzed for the $i^1$ antigen or blast-specific antigen and cells were assigned to a position in the cell cycle according to size and ability to incorporate $^3$H-thymidine into DNA. A majority of cell fractions from the rotor corresponding to the $G_1$ (or $G_0$) interval were negative for both specificities, whilst there was an enrichment of antigen-positive cells at the S and $G_2$ interval. This indicates that surface markers exist which recognize cells " in cycle ".

The above antigens are present on tumour cells, embryonic cells, and normal dividing cells; oncofoetal antigens have been demonstrated on tumour and embryonic cells, but as yet no attempt has been made to establish whether they are re-expressed on normal dividing cells. Are division antigens oncofoetal antigens? There still remains a need for "comparison . . . between the cancer cell and a normal cell growing equally rapidly ".

REFERENCES

Ben-or, S., Eisenberg, S. & Doljanski, F. (1960) *Nature, Lond.*, **188**, 1200.
Burger, M. M. & Goldberg, A. R. (1967) *Proc. natn. Acad. Sci. U.S.A.*, **57**, 359.
Cunniningham, D. D. & Pardee, A. B. (1969) *Biochemistry*, **64**, 1049.
Haddow, A. (1967) In *Cell Differentiation* CIBA Foundation Symposium. Ed. A. V. S. De Reuck and J. Knight.
Holley, R. W. (1972) *Proc. natn. Acad. Sci. U.S.A.*, **69**, 2840.
Hynes, R. O. & Bye, J. M. (1974) *Cell*, **3**, 113.
Kijmoto, S. & Hakamori, S. (1971) *Biochem. biophys. Res. Commun.*, **44**, 557.
Klein, G., Steiner, M., Wiener, F. & Klein, E. (1974) *Proc. natn. Acad. Sci. U.S.A.*, **71**, 685.
Purdom, L., Ambrose, E. J. & Klein, G. (1958) *Nature Lond.*, **181**, 1586.
Thomas, D. B. & Phillips, B. (1973) *J. exp. Med.*, **138**, 64.
Thomas, D. B. (1974) *Eur. J. Immunol.*, **4**, 819.